# Promoting Healthy Aging: Insights on Brain and Physiological Health - A Special Issue

**DOI:** 10.14336/AD.2023.0724

**Published:** 2023-08-01

**Authors:** Piotr Gronek, Yi-Yuan Tang

**Affiliations:** ^1^Department of Dance and Gymnastics, Faculty of Sport Sciences, Poznań University of Physical Education, Poland.; ^2^College of Health Solutions, Arizona State University, Phoenix, AZ 85004, USA

**Keywords:** healthy aging, brain, physical activity, nutrition, and stress management

## Abstract

This special issue focuses on healthy aging and neuroprotection, particularly in the context of brain and physiological health during normal aging and Alzheimer's disease. It highlights the importance of physical activity, nutrition, and stress management in promoting healthy aging and preventing neurodegenerative diseases. The issue explores molecular pathways, genetic factors, and lifestyle interventions that support brain and physiological health in aging populations. Overall, the findings presented in this special issue underscore the importance of healthy lifestyles in promoting brain and physiological health during the aging process.

The aim of this special issue is to provide insights on healthy aging. Research has shown that lots of factors affect healthy aging, from molecular to brain and physiology and to behavior and lifestyle. Among these factors, regular physical activity supports healthy aging including brain and physiological health and prevents aging-related disorders such as Alzheimer Disease (AD) [1, 2]. For example, studies have indicated that during the COVID-19 pandemic, lockdowns caused a decline in physical activity and an increase in sedentary lifestyle that contributed to the deterioration of brain and physiological health in the elderly [3]. Therefore, to maintain healthy aging, we need weekly physical activity recommended by the WHO. A recent review supported the resistance training recommendation in middle and late life, at a frequency of at least 3 sessions per week, to mitigate the neurological and cognitive consequences associated with aging, mainly through insulin-like growth factor-1 (IGF-1) [4]. Similarly, a recent study using drosophila melanogaster evaluated the anti-aging effects of probiotic (bacteria that provide a beneficial effect to the host) - Limosilactobacillus reuteri and also showed that the longevity effect of Limosilactobacillus reuteri is mediated by the reduction of the IGF-1 signaling pathway [5].

Moreover, during aging, many physiological processes occur such as cadiovascular system and respiratory system. Ribeiro et al provided an overview of the cardiac aging process with a focus on the following topics: cardiac structural and functional modifications, cellular mechanisms of cardiac dysfunction, genetics and epigenetics in the development of cardiac diseases and aging heart and response to the exercise [6]. These results sugegst that cardiac system is sensitive to physical activity and exercise during aging, consistent with the findings that long-term physical activity and exercise regulates cadiovascular system and reduces basal heart rate that may support brain and physiological health in aging population [7]. Aging processes also affect lung functions. The main alteration to lung function is the loss of lung elasticity with age. This process in the lungs increases vulnerability to respiratory infections due to the accumulation of cellular damage and senescence. Consequently, the lung environment undergoes major changes in mechanical function and other systemic factors. One review analyzed clinical studies to examine the influence of aging on respiratory infections. It turned out that respiratory infections pose a significant health problem among elderly, particularly during the COVID-19 pandemic. The increased mortality and morbidity rates among adults aged 65+ highlight the criticality of these infections. Therefore, it is important to prioritize prevention strategies, such as management of comorbidities through healthy lifestyles, to reduce the risk of lung infections in the elderly. For example, improving respiratory health through physical exercise and smoking cessation contributes to the risk reduction of lung infections in the elderly [8].

Nutrition is essential for maintaining overall health, especially for the elderly. Citicoline is a natural metabolite present in all living cells and breaks down to choline and cytidine when ingested. Choline is pivotal for learning and memory and one of the important constituents of neuronal membranes and myelin sheaths. Choline deficiency is correlated with memory dysfunction [9]. Studies showed that citicoline intake improves brain uptake of choline in elderly and may help in reversing early age-related cognitive decline. Similar effects of citicoline on memory indices are also found in patients with mild cognitive impairment and other neurological diseases. Altogether, these results suggest that oral citicoline intake positively influences memory function in humans who encounter age-related memory impairment and disease [9].

Chronic stress is another impaortant factor that sensitizes aging processes and AD. Growing evidence points to chronic stress as one of the major risk factors for aging and AD, as it can promote the onset and development of AD-related pathologies. Liu et al provided a window of opportunity for more effective preventive and identifying therapeutic strategies for stress-induced AD [10].

Amyloid beta (Aβ) and tau pathology have been shown in pathogenesis of AD, but the exact molecular mechanisms of AD remain elusive. Using caenorhabditis elegans model, an interesting report showed that the overexpression of activating transcription factor 7 (ATF7) significantly suppressed aging biomarkers and extended lifespan, suggesting that ATF7 is a longevity-promoting factor that lowers cellular senescence and inflammation in long-lived individuals [11]. Simialrly, a narrative review propsoed the activity of the cGAS-STING pathway in age-related diseases, discussed its general mechanisms in the onset and progression of age-related diseases and outlined the treatments targeting the cGAS-STING pathway for a potential therapeutic alternative for age-related diseases [12].

Many risk factors such as gene mutations associate with the onset of AD. Apolipoprotein E (APOE) is a well-established genetic risk factor for late onset AD and involved in regulating Aβ and tau pathology, inflammation, vascular integrity, glucose metabolism and vascular endothelial growth factor signaling. Importantly, growing evidence also indicates the role of APOE in retinal neurodegenerative diseases such as macular degeneration and glaucoma [13]. These findings suggest the overlapping molecular pathways in AD and age related retinal diseases. Relatedly, Ou-Yang et al summarized the molecular regulation mechanism of microglial autophagy in the AD pathology [14]. Accumulating evidence has shown that impaired microglial autophagy exerts considerable detrimental impact on the brain, including the inflammatory response, defective clearance, propagation of Aβ and Tau, and synaptic dysfunction, thus contributing to AD pathogenesis. These results support the roles of microglial receptors in autophagy regulation during AD and may shed lights on the potential drug development [14].

Taking the prevalence of neurodegenerative Parkinson's disease (PD) as an example, a recent review targets the prodromal period of neurodegenerative diseases and proposes early lifestyle modifications to reduce risk facotrs and alter disease progression [15]. For instance, the prodromal period of PD spans from those experiencing subtle motor deficits yet not meeting full diagnostic criteria or those with physiologic markers of disease alone. Therefore, identifying the early population is essential. Once identified, these patients could then potentially benefit from lifestyle modifications to alter the disease trajectory. While some prodromal symptoms substantially increase the risk of incident PD, others do not. Authors propose a multimodal approach to identify this group and stratify the risks, and then offer several combining strategies and tools to support overall health. It concludes that the preventions and modifications of neurodegenerative PD must be taken as earliest as possible to reduce the risks and symptoms [15].

In conclusion, this special issue mainly focuses on the brain and physiology (heart, lung, retinal) health during normal aging and AD stage. Healty lifestyles such as physical exercise, nutrition, reduced stress and substance use support healthy aging and prevention and treatment of neurodegenerative diseases. Future research should consider using biomarkers to personalize prevention and intervention in aging.
